# Complete Genomes of Two Xanthomonas translucens pv. translucens Strains Isolated from Barley in North Dakota

**DOI:** 10.1128/mra.00010-22

**Published:** 2022-03-28

**Authors:** Jeffrey K. Schachterle, Gongjun Shi, Thomas Baldwin, Zhaohui Liu

**Affiliations:** a USDA-ARS Cereal Crops Research Unit, Edward T. Schafer Agricultural Research Center, Fargo, North Dakota, USA; b Department of Plant Pathology, North Dakota State University, Fargo, North Dakota, USA; University of Arizona

## Abstract

Xanthomonas translucens causes the disease bacterial leaf streak in several cereal crops and grasses. Here, we report the complete genome sequences of two isolates of X. translucens pv. translucens that were isolated from barley in an important cereal crop production region.

## ANNOUNCEMENT

Xanthomonas translucens causes bacterial leaf streak disease in a variety of cereal crops and grasses, with distinct pathovars with different host ranges (1–3). These pathovars include the economically significant pathovars X. translucens pv. undulosa and X. translucens pv. translucens (4). Isolates from X. translucens pv. undulosa have a broader host range and cause disease on major cereal crops, including wheat and barley; in contrast, X. translucens pv. translucens isolates primarily infect barley (reviewed in reference 5). Due to limited genome sequences for isolates representing X. translucens pv. translucens, here we present complete genomes of the bacterial leaf streak X. translucens pv. translucens strains XttB1FA and XttB8GF. These two strains represent the X. translucens pv. translucens population from a major barley production region in the United States. The sequences of these isolates will serve as resources for understanding the biology of X. translucens pv. translucens and host-pathogen interactions between X. translucens pv. translucens and barley.

The strains XttB1FA and XttB8GF were isolated in 2017 from naturally infected barley fields in eastern North Dakota. Bacterial strains were restreaked from single-colony-derived cultures that had been stored in a −80°C freezer and were grown on Wilbrink’s agar medium at 28°C. Bacterial cells were collected after 2 days of culturing, and DNA was isolated following the method of Richards et al. (6) with modifications. Briefly, 350 mg of cells (wet weight) was suspended in lysis buffer, treated with RNase A (6), and incubated for 45 min at 60°C. Proteinase K was added to a final concentration of 0.24 mg/mL, and samples were incubated for an additional 30 min at room temperature. Samples were treated with potassium acetate, extracted with chloroform, and precipitated by the addition of isopropyl alcohol. Pellets were solubilized in 500 μL of sterile water. Library preparation and sequencing were conducted by the Genome Analysis Core, Mayo Clinic (Rochester, MN), using the PacBio Sequel platform (Pacific Biosciences, Menlo Park, CA) with a 10-kb insert size. Each strain was run on its own single-molecule real-time (SMRT) cell, generating a total of 21,702 reads, with an *N*_50_ value of 10,962 bp, and 17,468 reads, with an *N*_50_ value of 10,486 bp, and yielding 6.25 and 8.97 Gb for XttB1FA and XttB8GF, respectively. Default parameters were used for genome assembly and annotation software unless otherwise specified. Reads were assembled using Canu v2.0 (7); the assembly for XttB1FA is 4,661,643 bp in length, and that for XttB8GF is 4,708,077 bp, both as single complete contigs (Table 1).

**TABLE 1:** Features of X. translucens pv. translucens genomes

Strain	Isolation host	Isolation country	Year of isolation	Genome size (bp)	GC content (%)	No. of coding sequences	No. of TALEs
XttB1FA	Barley	USA	2017	4,661,643	67.9	3,933	5
XttB8GF	Barley	USA	2017	4,708,077	67.9	4,006	5
UPB886	Barley	Iran	1990	4,674,364	67.9	3,926	5

**FIG 1:**
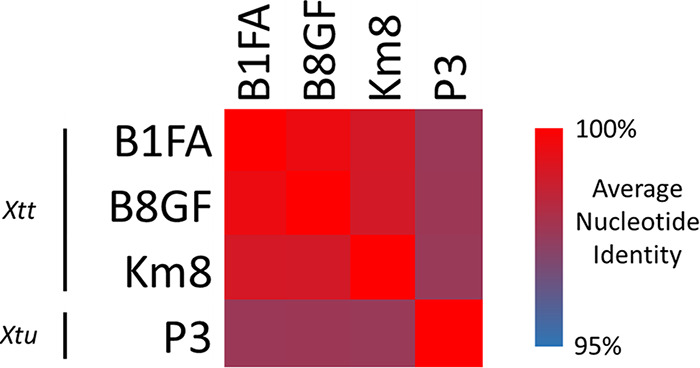
XttB1FA and XttB8GF genomes show high ANIs with each other and with another X. translucens pv. translucens (*Xtt*) strain, Km8. All of the X. translucens pv. translucens strains have lower ANIs with the X. translucens pv. undulosa (*Xtu*) strain P3 than with each other. ANIs between strains were calculated using the OrthoANIu method of Yoon et al. (10) via their online webserver (https://www.ezbiocloud.net/tools/ani) with default parameters.

Annotations were conducted using the NCBI Prokaryotic Genome Annotation Pipeline (PGAP) (8), resulting in 3,933 and 4,006 predicted protein coding sequences for XttB1FA and XttB8GF, respectively. Like the recently published UPB886 genome (9), both the XttB1FA and XttB8GF genomes are predicted to encode 5 transcription activator-like effector (TALE) proteins (Table 1). Both the XttB1FA and XttB8GF genomes are predicted to encode an additional 20 predicted type III secretion effector proteins. The XttB1FA and XttB8GF genomes shared high average nucleotide identity (ANI) with each other and higher ANIs with Km8, another X. translucens pv. translucens strain, than with P3, an X. translucens pv. undulosa strain (Fig. 1).

These new genome sequences will facilitate comparative analyses for the identification and characterization of effectors and other genetic elements governing differences in host specificity and virulence between isolates in the X. translucens pv. translucens and X. translucens pv. undulosa groups.

### Data availability.

The complete genome sequences have been deposited in GenBank and are available under BioProject accession number PRJNA789891 with accessions numbers CP090000 (XttB1FA) and CP089999 (XttB8GF). The raw reads have also been deposited in the NCBI database as fastq files (SRA accession numbers SRX14353264 [XttB1FA] and SRX14353265 [XttB8GF]).
